# Therapeutic Potential of Arimoclomol Nanomicelles: In Vitro Impact on Alzheimer’s and Parkinson’s Pathology and Correlation with In Vivo Inflammatory Response

**DOI:** 10.1021/acschemneuro.4c00734

**Published:** 2025-02-05

**Authors:** Isabelle Xavier-de-Britto, Natália Cristina Gomes-da-Silva, Marilia Amável Gomes Soares, Cristian Follmer, David Dabkiewicz, Luciana Magalhães Rebelo Alencar, Celso Sant’Anna, Tatiana Paula Teixeira Ferreira, Patrícia Machado Rodrigues e Silva Martins, Eduardo Ricci-Junior, Pierre Basílio Almeida Fechine, Ralph Santos-Oliveira

**Affiliations:** † Brazilian Nuclear Energy Commission, Nuclear Engineering Institute, Laboratory of Nanoradiopharmacy and Synthesis of New Radiopharmaceuticals, Rio de Janeiro, Rio de Janeiro 21941906, Brazil; ‡ Laboratory of Biological Chemistry of Neurodegenerative Disorders, Department of Physical Chemistry, Institute of Chemistry, Federal University of Rio de Janeiro, Rio de Janeiro 21941-909, Brazil; ¶ Group of Chemistry of Advanced Materials (GQMat)–Department of Analytical Chemistry and Physical-Chemistry, Federal University of Ceará, Fortaleza, Ceará 451-970, Brazil; § Biophysics and Nanosystems Laboratory, Federal University of Maranhão, Department of Physics, São Luis, Maranhão 65065690, Brazil; ∥ Laboratory of Microscopy Applied to Life Science–Lamav, National Institute of Metrology, Quality and Technology, Duque de Caxias, Rio de Janeiro 25250-020, Brazil; ⊥ Laboratory of Inflammation, Oswaldo Cruz Institute, Oswaldo Cruz Foundation, Rio de Janeiro 21040-360, Brazil; # Federal University of Rio de Janeiro, School of Pharmacy, Rio de Janeiro, Rio de Janeiro 21941900, Brazil; ∇ Rio de Janeiro State University, Laboratory of Radiopharmacy and Nanoradiopharmaceuticals, Rio de Janeiro 23070200, Rio de Janeiro, Brazil

**Keywords:** drug delivery, neurodegenerative disease, inflammation

## Abstract

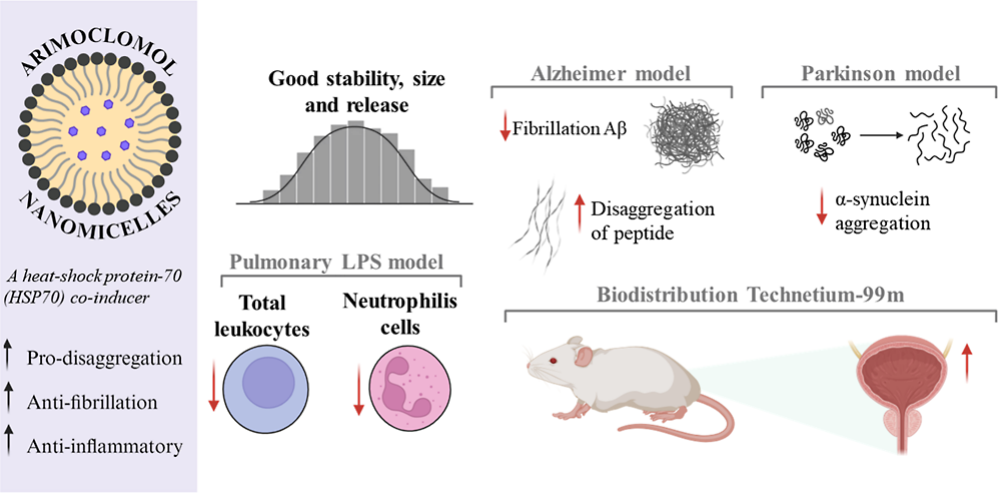

This study investigates the potential of arimoclomol-loaded nanomicelles for the treatment of neurodegenerative diseases like Alzheimer’s and Parkinson’s, as well as their anti-inflammatory properties. Arimoclomol, a coinducer of heat shock proteins (HSPs), has shown clinical promise in mitigating protein misfolding, a hallmark of these diseases. In this work, arimoclomol nanomicelles significantly reduced the aggregation of β-amyloid (Aβ_1–42_) and α-synuclein (α-syn), key pathological proteins in Alzheimer’s and Parkinson’s. Additionally, the nanomicelles demonstrated potent anti-inflammatory effects, reducing leukocyte and neutrophil counts in an acute inflammation model. These results suggest that arimoclomol nanomicelles could enhance clinical outcomes by targeting both neurodegenerative and inflammatory processes, offering a promising therapeutic strategy for long-term disease management.

## 1 Introduction

The central nervous system (CNS) undergoes a degenerative process marked by a progressive loss of neuronal integrity. This degeneration is primarily driven by a breakdown in the connectivity and communication between neurons, which are crucial for sensory, motor, and cognitive functions, including vision, hearing, movement, speech, language, and memory. The process is characterized by the gradual degradation of synapses and axons, ultimately leading to neuronal death.^1,2^ Aging is the primary risk factor for most neurodegenerative diseases, such as Alzheimer’s and Parkinson’s. However, several other factors also contribute significantly, including gender, unhealthy lifestyle habits, vascular conditions, genetic predispositions, and more recently identified factors like hearing loss.^3,4^

In AD, the disease is primarily characterized by the accumulation of β-amyloid (Aβ) and tau proteins, which leads to the formation of neurofibrillary tangles. The progression of AD is gradual, typically beginning with cognitive impairment and memory loss, indicative of its heterogeneous biological nature. The pathophysiology involves the aggregation of Aβ protein, which results from the selective cleavage of amyloid precursor protein (APP).^5^ Under normal physiological conditions, APP is cleaved by α-secretase, which prevents the formation of Aβ fragments, followed by subsequent cleavage by γ-secretase. However, under pathological conditions, APP is cleaved by β-secretase, leading to the release of the sAPPβ fragment, contributing to the formation of Aβ plaques.^6,7^

Tau protein, a microtubule-associated protein, also plays a crucial role in AD pathophysiology. In the human brain, tau exists in six isoforms produced through alternative splicing. Under normal circumstances, tau is soluble and natively unfolded, interacting with tubulin to facilitate its assembly into microtubules, thereby stabilizing the microtubule structure. In the brains of AD patients, tau becomes hyperphosphorylated at multiple serine, threonine, and tyrosine residues due to an imbalance in the activities of tau kinases and phosphatases, leading to the formation of insoluble tau aggregates that disrupt neuronal function.^8−10^

In contrast, Parkinson’s disease (PD) is characterized by the pathological aggregation of the α-synuclein protein (α-syn), which triggers selective and progressive neuronal death. This aggregation contributes to mitochondrial impairment, lysosomal dysfunction, synaptic alterations (including interactions with dopamine transporters), and disruptions in calcium homeostasis. α-Syn is a neuronal protein composed of 140 amino acids and is involved in regulating cell differentiation, synaptic plasticity, and dopaminergic neurotransmission. Its abnormal aggregation plays a central role in the degeneration of dopaminergic neurons characteristic of PD.^11,12^

Currently, approximately 6.9 million people in the United States are living with Alzheimer’s disease. By 2050, this number is projected to nearly double, reaching around 13 million.^13^ In 2020, over 55 million people worldwide were living with some form of dementia. This figure is expected to increase to 78 million by 2030 and further escalate to 139 million by 2050.^14^

The prevalence of Parkinson’s disease (PD) has doubled over the past 25 years. In 2019, data indicated that approximately 8.5 million people were diagnosed with PD globally, representing an 81% increase compared to the early 2000s. According to the Parkinson’s Foundation’s Parkinson’s Prevalence Project, the number of individuals living with Parkinson’s disease in the United States is projected to reach 1.2 million by 2030.^15,16^

The cost of providing care for individuals with Alzheimer’s disease and other dementias is estimated to reach approximately $360 billion in 2024. Projections suggest that this expense could escalate to nearly $1 trillion (in today’s dollars) by 2050.^17^ In 2017, the total economic burden of Parkinson’s disease (PD) in the United States was estimated at $52 billion, with $25.4 billion of this amount attributed to direct medical costs.^18^ Inflammation plays a significant role in the pathophysiology of both Alzheimer’s disease (AD) and Parkinson’s disease (PD), contributing to the progression and severity of these neurodegenerative disorders.

In AD, the accumulation of β-amyloid (Aβ) plaques and hyperphosphorylated tau protein tangles in the brain triggers a chronic inflammatory response. Microglia, the resident immune cells of the central nervous system (CNS), recognize Aβ plaques as foreign or damaged structures, leading to their activation.^19^ Once activated, microglia release pro-inflammatory cytokines (e.g., IL-1β, IL-6, and TNF-α), chemokines, and reactive oxygen species (ROS). This inflammatory milieu exacerbates neuronal damage and promotes further Aβ aggregation, creating a vicious cycle of inflammation and neurodegeneration. Additionally, astrocytes, another type of glial cell, become reactive in AD, contributing to the chronic inflammatory state and further promoting neuronal dysfunction.^20,21^

In PD, inflammation is also a key player in disease progression. The aggregation of α-synuclein (α-syn) within neurons leads to the activation of microglia and astrocytes, which secrete pro-inflammatory cytokines and ROS. This neuroinflammatory response contributes to the degeneration of dopaminergic neurons in the substantia nigra, a hallmark of PD. Moreover, evidence suggests that systemic inflammation, such as elevated levels of pro-inflammatory cytokines in the blood, may exacerbate neuroinflammation in PD.^19,22^ Chronic inflammation in PD is associated with increased oxidative stress and mitochondrial dysfunction, which further promote α-syn aggregation and neuronal death.^22^

Arimoclomol is a hydroxylamine derivative that acts as a coinducer of the cellular heat shock response by enhancing the production of Heat Shock Protein 70 (HSP70).^23,24^ HSP70 belongs to a highly conserved family of proteins that are crucial for maintaining cellular homeostasis, especially under stress conditions. As part of the broader heat shock protein (HSP) family, HSP70 is upregulated in response to various stressors, including elevated temperatures, toxins, and oxidative stress,^25^ playing a pivotal role in protein folding, preventing protein aggregation, and assisting in the repair or degradation of damaged proteins.^26^

The application of nanotechnology offers innovative solutions to the challenges associated with targeting Heat Shock Protein 70 (HSP70) more precisely and effectively in the treatment of neurodegenerative and inflammatory diseases. Nanoparticles can be engineered to deliver therapeutic agents directly to specific cells or tissues, significantly reducing the risk of systemic side effects and enhancing treatment efficacy. By encapsulating HSP70-inducing compounds, such as arimoclomol, within nanoparticles, it is possible to achieve targeted delivery to the brain or other affected areas, thereby optimizing therapeutic outcomes while minimizing exposure to healthy tissues or tissues susceptible to cancer.

In this study, we have successfully developed, fully characterized, and evaluated arimoclomol-loaded nanomicelles through in vitro and in vivo experiments as a potential therapeutic strategy for treating inflammatory and neurodegenerative diseases. This approach offers a promising avenue for enhancing the therapeutic potential of arimoclomol by improving its bioavailability, targeting specificity, and overall efficacy in disease management.

## 2 Materials and Methods

### 2.1 Reagents

All reagents and solvents used in this study were purchased from Sigma-Aldrich (Brazil), with exception to the arimoclomol that has been acquired from APIChem-Research chemicals.

### 2.2 Arimoclomol Nanomicelles

A concentration of 30 mg of arimoclomol was added to Pluronic F127 (20% w/v). The system was gently stirred for 1 min and then processed for 2 min using an ultrasonic processor (UP100H, Hielscher, power: 100%, cycle: 1) at 2 °C using a thermocycler (SolidSteel model SSDu).

### 2.3 Atomic Force Microscopy (AFM)

#### 2.3.1 Sample Preparation

The particle solutions were diluted to approximately 10^9^ to 10^10^ particles per 1 cm^3^. The solutions were dropped in fresh cleaved mica and left to dry in a vacuum chamber protected from contamination.

#### 2.3.2 AFM Setup

Atomic force microscopy (AFM) experiments were performed with a Multimode 8 (Bruker, Santa Barbara) using Nanoscope software (Bruker) in PeakForce Quantitative Nanomechanics (QNM) mode. The experiments were performed with a cantilever spring constant of 0.4 N/m and a nominal tip radius of 2 nm. The experiment was performed with a scan resolution of 256 × 256 lines and a scan frequency of 0.5 Hz. The images were analyzed using Gwyddion 2.60 software and Nanoscope Analysis 2.0. For the particle diameter analysis, AFM maps of 10 μm containing hundreds of nanoparticles were analyzed in Nanoscope Analysis in particle analysis mode, and the mean diameter of lamivudine nanoparticles was calculated.

### 2.4 Raman Spectroscopy

To evaluate the encapsulation efficiency, Raman spectroscopy was employed. The spectrometer was calibrated for the wavenumber scale using cyclohexane (Vetec/Sigma-Aldrich, purity >99%) as a reference material. The band positions are referenced according to ASTM E1840-96 (2022). The samples were deposited onto a glass slide, which was placed beneath the objective of the Raman microscope. Five spectra were collected from different points on the sample. The assays were conducted using a Witec Alpha A/R spectrometer operating in micro-Raman mode with a backscattering configuration. The excitation source was a 532 nm (2.33 eV) solid-state laser, and a 50× objective (NA 0.80) was used. The laser power was set to less than 1 mW (measured at the objective output). The spectral region analyzed was from 0 to 3500 cm^–1^, with a diffraction grating of 600 mm^–1^. Finally, the acquisition time per spectrum was 30 s at a temperature of 21.0 ± 0.3 °C, with a relative humidity of 54 ± 1%.

### 2.5 Release Profile

The release profile of the drug was analyzed using a dialysis bag method. Initially, the dialysis bag was immersed in distilled water to ensure proper hydration of the membrane. Subsequently, 1 mL of arimoclomol nanomicelles at a concentration of 15 mg/mL was introduced into the hydrated dialysis bag. This prepared bag, containing the arimoclomol nanomicelles, was then placed in a beaker filled with 0.2 L of distilled water. The beaker was maintained at a constant temperature of 37 °C and stirred at a rate of 100 rpm, utilizing an IKA C-MAG HS 7 magnetic stirrer equipped with heating capabilities. Sampling for analysis occurred at predetermined intervals: 0 to 72 h. At each time point, 4 mL of the solution (in triplicate) was extracted from the beaker for UV–vis spectroscopy (Kasvi, K37-UVVIS) analysis. It is critical to note that each time a sample was removed for analysis, an equivalent volume of distilled water was replenished to maintain a consistent volume throughout the experiment. Finally, the samples were read in UV–vis spectrophotometry at a wavelength of 255 nm and processed in GraphPad Prism 8.0 and the statistic used was the standard deviation (SD).

### 2.6 In Vitro: Fibrillation and Fibril Dissolving Assay

#### 2.6.1 Expression and Purification of Recombinant α-Synuclein and Aβ_1–42_ Monomers

The expression and purification of α-synuclein were carried out as described.^27^ The molar concentration of α-synuclein was determined by measuring the absorbance at 276 nm with a molar extinction coefficient of 5600 M^–1^ cm^–1^. For the expression and purification of Aβ_1–42_ peptide, the plasmid pET vector [pET-Sac-Abeta (M1–42)] was transformed into BL21(DE3) pLysS and the peptide was purified based on a previously described procedure.^28^ The molar concentration of the Aβ_1–42_ monomer was determined by measuring absorbance at 276 nm with a molar extinction coefficient of 1460 M^–1^ cm^–1^.

#### 2.6.2 Evaluation of the Fibril-Destabilizing and Antifibrillogenic Activities of Arimoclomol Nanomicelles or Arimoclomol

Fibrilation of α-synuclein/Aβ_1–42_ was done by incubation of 100 μM of the monomer in either 10 mM sodium phosphate, pH 7.4, 100 mM NaCl (for α-synuclein) or 20 mM HEPES, pH 6.5 (for Aβ_1–42_) at 37 °C under agitation (450 rpm). The formation of fibrils was confirmed by mixing the protein solution with 5 μM thioflavin-T (ThT) and measuring the fluorescence emission at 485 nm upon excitation at 446 nm using a Cary Eclypse Fluorimeter (Agilent Technologies, Santa Clara, USA). To analyze the fibril-destabilizing activity, 25 μM of preformed fibrils of either α-synuclein or Aβ_1–42_ (10 mM sodium phosphate, pH 7.4, 100 mM NaCl plus 5 μM ThT) were incubated in the presence of 50, 100, or 200 μM of arimoclomol nanomicelles or an equivalent concentration of Pluronic F127 (nanomicelles control), and the ThT fluorescence monitored in course of the time at 25 °C, no agitation. In case of arimoclomol (dissolved in 100% DMSO), an equivalent concentration of DMSO was used as the control. To evaluate the inhibitory activity of arimoclomol nanomicelles or arimoclomol on the fibrillation of α-synuclein and Aβ_1–42_, 70 μM of the protein monomer, in the presence of 5 μM of ThT, were incubated in the presence of 200 μM of arimoclomol nanomicelles (or nanomicelles control) or arimoclomol (or DMSO) at 37 °C, 450 rpm, in a 96-well microplate using thermomixer equipment (Eppendorf, Hamburg, Germany).

### 2.7 In Vivo Biodistribution: Tissue Deposition and Inflammatory Assay

#### 2.7.1 Labeling Process with ^99m^Tc

The process of labeling with technetium-99 commenced by adding 1 mL of stannous chloride (SnCl_2_) at a concentration of 80 μL/mL (obtained from Sigma-Aldrich), combined with 1 mL of technetium-99m (^99m^Tc) with an activity of 544 μCi, followed by a 10 min incubation period. Subsequently, 15 mg of arimoclomol nanomicelles was incorporated into this mixture and subjected to a further 10 min incubation to label the structures.

#### 2.7.2 Quality Control of the Labeling Process with Tc-99m

To confirm the efficacy of the labeling process, Radio Thin Layer Chromatography (RTLC) was done using Whatman paper no 1 using 2 μL of the ^99m^Tc-arimoclomol nanomicelles and acetone (Sigma-Aldrich) as mobile phase at times of 0, 1, 2, 4, 6, and 22 h. The radioactivity of the strips was verified in a γ-counter (Hidex, Turku, Finland). The RTLC was performed in triplicate for each time.

#### 2.7.3 Animals

Experiments were performed on Balb/c or Swiss–Webster mice, male, *n* = 4, weighting between 25 and 30 g. Animals were housed one per cage under controlled conditions of luminosity (12:12 h light and dark cycle) and temperature (21.0 ± 1.0 °C), with free access to water and standard chow. All procedures were approved by the State University of Rio de Janeiro Animal Care and Use Committee (Rio de Janeiro, RJ, Brazil; protocol number CEUA/8059100220/2021 and CEUA/IOC/FIOCRUZ L-001/2019-A3), which is consistent with United States National Institute of Health Guide for Care and Use of Laboratory Animals (National Research Council, 1996).

#### 2.7.4 Animal Preparation

Animals were anesthetized by an intraperitoneal injection (ketamine 100 mg kg^–1^ and xylazine 20 mg kg^–1^).

#### 2.7.5 Design Protocol

For the biodistribution/tissue deposition studies, 27.2 μCi (1.0 MBq)/0.2 mL of ^99m^Tc-nanomicelles was injected intraperitonially (i.p.), evaluating the systemic behavior in healthy animals. Animals were sacrificed 24 h postinjection, by using excess of anesthesia (isoflurane chamber), the blood and organs of interest heart, brain, stomach, intestine, bladder, kidney (right and left), lung (right and left), liver, spleen were immediately dissected out and weighed for quantitative estimation of gamma counts using a gamma counter (Hidex, Turku, Finland). Results were expressed as percentage of injected dose per organ (% ID/g).

#### 2.7.6 LPS-Induced Lung Injury

Swiss–Webster mice were anesthetized with a mixture of isofluorane (0.5%) and atmospheric air by oropharyngeal aspiration of lipopolysaccharide (LPS) (25 μg/25 μL) (from *Escherichia coli* serotype 0127:B8; SIGMA, St. Louis, MO, USA) or sterile 0.9% saline, and the analyses were made 24 h later. For the analyses of cells infiltrated into the airways, animals were sacrificed with ketamine (300 mg/kg) and xylazine (30 mg/kg) and the bronchoalveolar lavage fluid (BALF) performed by means of twice 750 μL of PBS with ethylenediamine tetra-acetic acid (EDTA, 10 mM). BALF was retrieved and centrifuged (3000 rpm, 4 °C for 10 min) and the cell pellet resuspended in EDTA–PBS (250 μL). Total leukocytes were enumerated by means of Neubauer chamber and Türk solution. The differential analyses were performed in cytocentrifuged smears stained with May-Grunwald-Giemsa dye, under an oil immersion objective and light microscopy (Olympus BX50), and the final counts were reported as number of cells (×105) per BALF. Animals were treated with arimoclomol nanomicelles, at a dose of 100 mg/kg, administered intraperitoneally (i.p.), 1 h before LPS stimulation. The same volume of vehicle was administered into the control groups. Animals were divided into 3 experimental groups, consisting of at least 6 animals: (i) saline-stimulated; (ii) LPS-stimulated and treated with vehicle; (iii) LPS-stimulated and treated with arimoclomol nanomicelles.

### 2.8 Radiopharmacokinetic (PK) Analysis

For radiopharmacokinetics studies, the Balb/c mice received 7.5 mg of radiolabeled arimoclomol nanomicelles (^99m^Tc-arimoclomol nanomicelles) administered by intraperitoneal injection. Subsequently, 2 μL of blood samples were collected from the tail vein at the following time points: 0, 1, 2, 3, 18, 22, 23, 22 and 24 h. The calculated pharmacokinetic parameters were: (i) zero-time concentration (*C*_0_), (ii) elimination constant (*K*_e_), (iii) volume distribution (*V*_d_), (iv) clearance (CL), and (v) half-life elimination (*t*_1/2_). The radioactive count conversion to arimoclomol nanomicelles mass was calculated considering the initial mass of 7.5 mg using the eq 1.
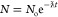
1

### 2.9 Biochemistry Analysis

Blood specimens were obtained through cardiac puncture from healthy mice that received an intraperitoneal injection of arimoclomol nanomicelles, comprising the intervention group, 24 h after administration (with a sample size of *n* = 3 per group). Subsequently, 0.5 mL of these blood samples were transferred into microtubes prefilled with 0.5 mL of the anticoagulant Heparin (sourced from Sigma-Aldrich, Brazil). To separate the plasma, the samples underwent centrifugation at 5000 rpm for 5 min at a temperature of 4 °C. Following separation, the plasma samples were processed in alignment with the protocols provided by the manufacturer (Bioclin, MG, Brazil). This processing aimed to assess the enzymatic activities of several key biomarkers: alanine aminotransferase (ALT), aspartate aminotransferase (AST), gamma-glutamyl transferase (GGT), creatinine (CRE), lactate dehydrogenase pyruvate (LDH-P), glucose (GLU), lipase D (LPS) and amylase (AMS), providing insights into the physiological impact of the arimoclomol nanomicelles treatment on the mice.

### 2.10 Statistical Analysis

The data obtained from the cell viability assay was plotted in the GraphPad Prism 8.1 program. The experiments were carried out at least three times with six experimental replicates. The data was analyzed by one way ANOVA to determine the difference between the different groups and the control. Statistical significance is shown by the asterisks. *p < 0.05 was considered significant, **p < 0.01 was considered highly significant, and ***p < 0.001 was considered very highly significant.

## 3 Results

### 3.1 Atomic Force Microscopy (AFM)

**Figure 1:**
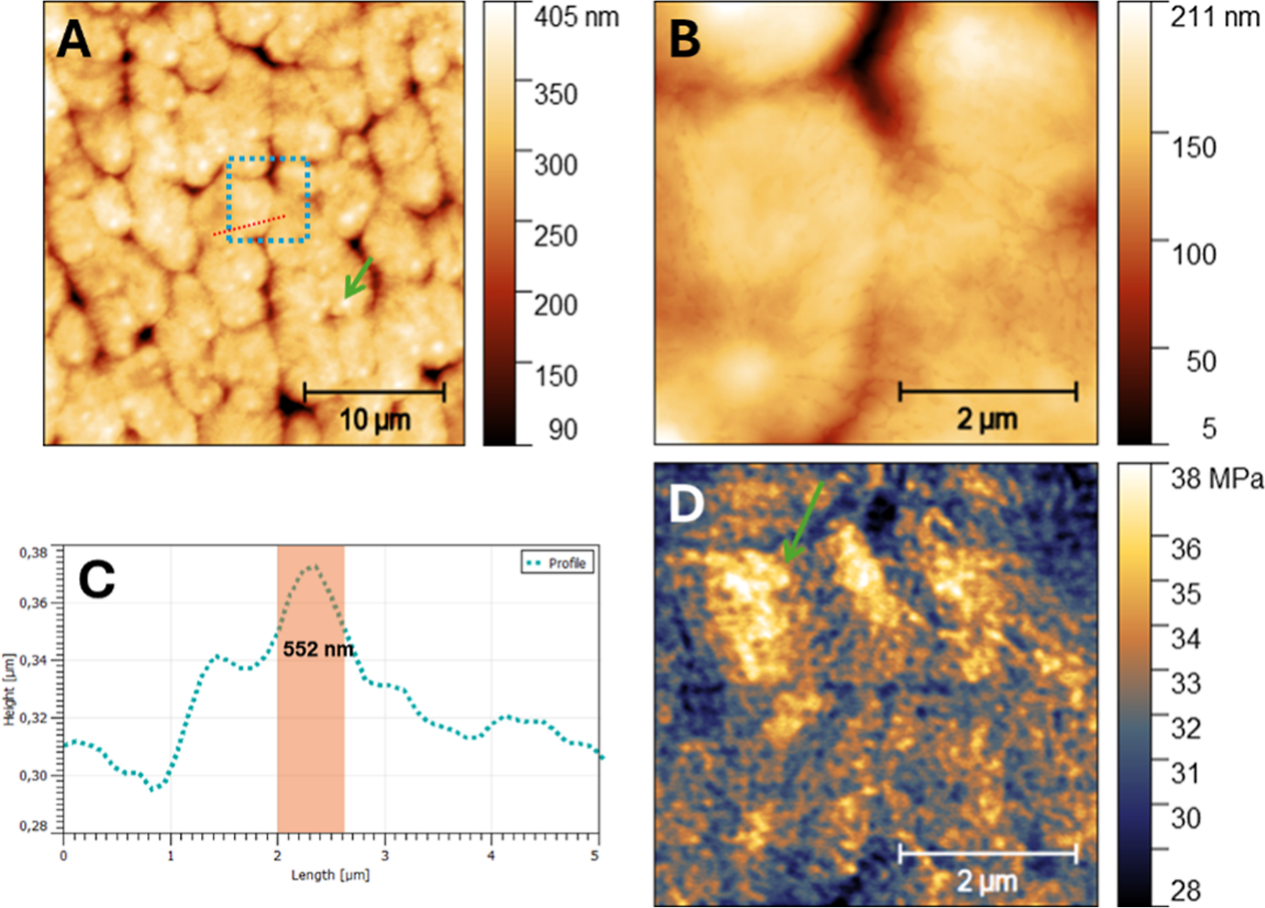
Atomic force microscopy for the characterization of arimoclomol nanomicelles. (A) Morphological image of the micellar structures, with calibration bar at 10 μm. (B) Morphological image of the arimoclomol micelles with a 2 μm bar. (C) Measurement of the diameter of the nanomicelles characterized by the red line. The green arrows show the presence of arimoclomol associated with the micellar structures.

The AFM results of the micellar film are shown in Figure 1. The micellar structures (Figure 1A) present a morphological image of the micellar structures, with a calibration bar at 10 μm. This structure possibly participates in the stabilization of the micellar structure. In Figure 1B it is possible to observe a morphological image of the arimoclomol micelles with a bar of 2 μm. And in Figure 1C it shows the measurement of the diameter of the characterized nanomicelles and the presence of arimoclomol associated with the micellar structures.

### 3.2 Raman Spectroscopy

**Figure 2:**
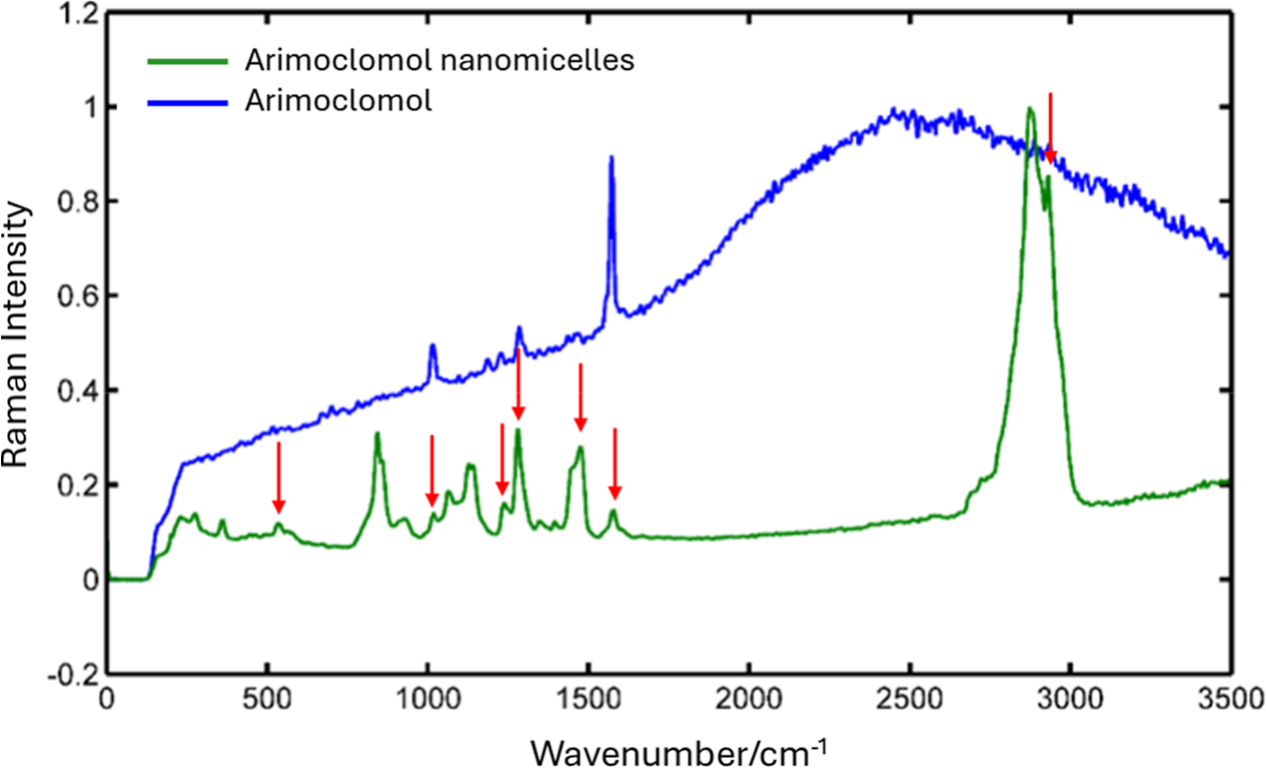
Raman spectra comparing free arimoclomol (blue) and arimoclomol-loaded nanomicelles (green). Key vibrational peaks of arimoclomol are marked by red arrows, indicating distinct shifts in the nanomicelle spectrum. These shifts suggest successful encapsulation of arimoclomol within the nanomicelles, altering its molecular environment and vibrational properties.

The representative spectra of the samples are shown in Figure 2. The maximum intensity of the graphs has been normalized for better visualization. The spectra obtained underwent a constant baseline removal process using the region between 0 and 100 cm^–1^ as a reference. The uncertainty in the wavenumber was estimated by combining the uncertainties relating to spectral resolution and calibration with MR. Assuming a coverage factor of *k* = 2, corresponding to a coverage probability of 95.45%, the maximum expanded uncertainty for the entire wavenumber range is *U* = 3.4 cm^–1^. The uncertainties have been reported in accordance with the publication Evaluation of Measurement Data.^29^

A peak at 2500 cm^–1^ is unusual for Raman spectroscopy, as it does not typically correlate with common vibrational modes (e.g., C–H, O–H, or N–H stretching). The broadening of the peak for free arimoclomol and its sharpening in nanomicelles suggests a change in molecular environment or interaction upon encapsulation (Supporting Information).

### 3.3 Release Profile

**Figure 3:**
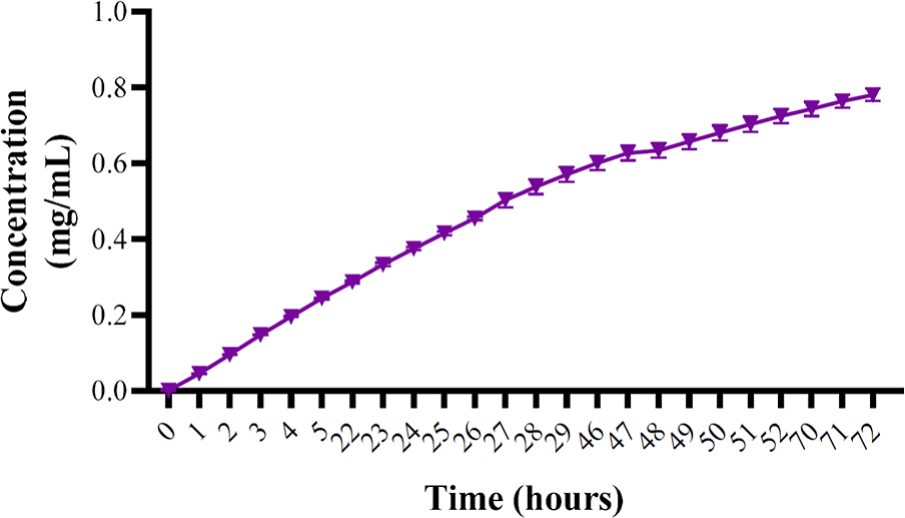
Release profile of arimoclomol nanoencapsulated in Pluronic F127 (20% w/v). The data show a controlled release by UV–vis spectrophotometry at a wavelength of 255 nm. The data was plotted in GraphPad Prism software and the standard deviation (SD) was taken into account.

T the release profile test, as depicted in Figure 3, demonstrated a rapid increase in the release of arimoclomol from the nanomicelles within the first hour, followed by a stable and sustained release up to 78 h. This release pattern indicates that the nanomicelles provide an initial burst release, ensuring a prompt therapeutic effect, and then maintain a consistent drug delivery over an extended period, which is advantageous for sustained therapeutic action in the treatment of neurodegenerative diseases.

### 3.4 In Vitro: Fibrillation Assay

#### 3.4.1 Disaggregation and Antifibrillogenic Test for Amyloid β

**Figure 4:**
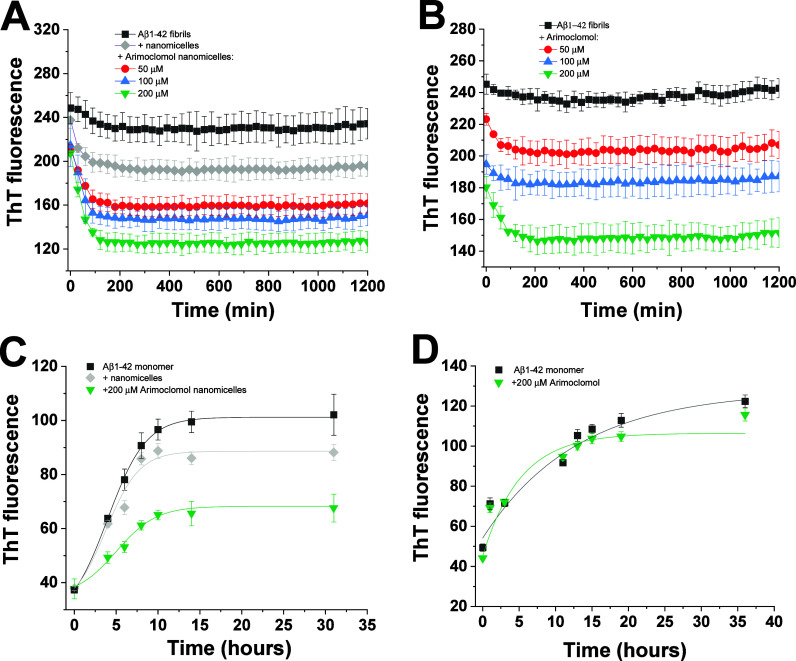
Fibril-destabilizing and antifibrillogenic activities of arimoclomol nanomicelles against Aβ_1–42_. Kinetics of disaggregation of fibrils of Aβ_1–42_ (25 μM) monitored by ThT fluorescence at 25 °C, no agitation, in the presence of 50, 100, or 200 μM of arimoclomol nanomicelles (A) or arimoclomol (B). Equivalent concentrations of Pluronic F127 (nanomicelles control) or DMSO were used as the control in A and B, respectively. Inhibition of the fibrillation of 70 μM Aβ_1–42_ monomer upon treatment with 200 μM of arimoclomol nanomicelles (C) or arimoclomol (D), at 37 °C, 450 rpm. Results mean values ± standard deviation from three (in A,B) or six (in C,D) independent experiments.

Thioflavin T (ThT) aggregation assay was employed to evaluate the impact of arimoclomol nanomicelles on the aggregation of β-amyloid (Aβ_1–42_) peptide. The Figure 4A present the disaggregation analysis of preformed fibrils of Aβ_1–42_ over time indicating that the treatment with arimoclomol nanomicelles led to a concentration-dependent reduction in the ThT signal compared to nanomicelles alone, which suggest a fibril-destabilizing activity of arimoclomol nanomicelles. Similar results were observed for arimoclomol solubilized in DMSO (B), even though it exhibited a diminished ability to dissolve Aβ_1–42_ fibrils compared with arimoclomol nanomicelles. For instance, at a concentration of 50 μM, arimoclomol nanomicelles reduced the ThT intensity approximately 35%, while a reduction of only 10% was observed at the same concentration of arimoclomol. This suggests that the nanomicelles possess a strong ability to disaggregate preformed Aβ_1–42_ fibrils. The Figure 4C shows that arimoclomol nanomicelles are also capable of inhibiting the fibrillation Aβ_1–42_ monomer. Interestingly, even at a concentration of 200 μM, arimoclomol solubilized in DMSO failed in inhibiting the fibrillation of Aβ_1–42_ compared with arimoclomol nanomicelles. Although these are preliminary results, they highlight the potential of arimoclomol nanomicelles as a therapeutic agent capable of both disaggregating existing Aβ_1–42_ fibrils and inhibiting their formation, offering promising insights into their application in the treatment of Alzheimer’s disease.

#### 3.4.2 Effect of Arimoclomol Nanomicelles on α-Synuclein Aggregation

**Figure 5:**
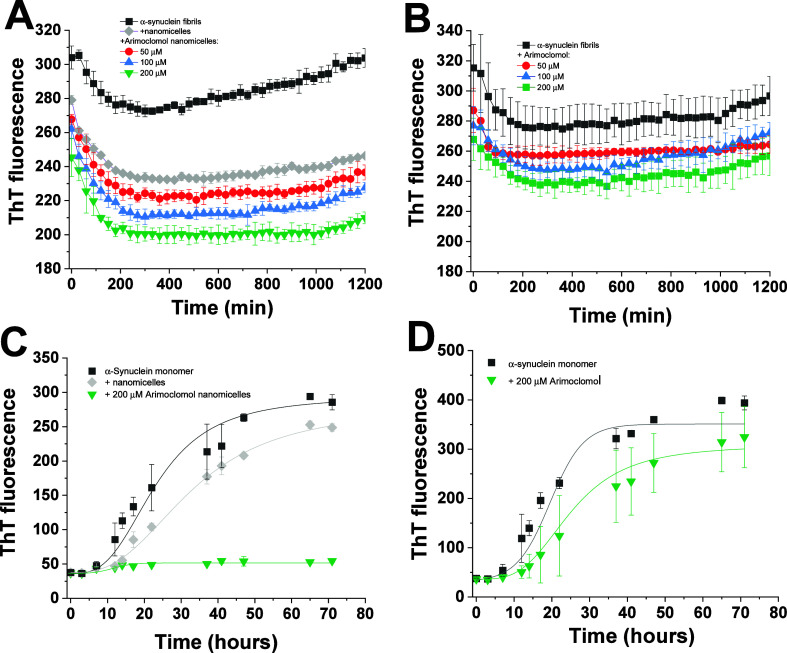
Effect of arimoclomol nanomicelles on either fibril dissolving or inhibition of fibrillation of α-synuclein. Panels A and B shows ThT fluorescence intensity of samples of fibrils of α-synuclein (25 μM) at 25 °C, no agitation, in the presence of 50, 100, or 200 μM of arimoclomol nanomicelles or arimoclomol, respectively. Inhibitory effect of 200 μM arimoclomol nanomicelles (C) or arimoclomol (D) on the fibrillation of 70 μM α-synuclein monomer at 37 °C, 450 rpm. Results mean values ± standard deviation from three (in A,B) or six (in C,D) independent experiments.

We extended our investigation to assess the impact of arimoclomol nanomicelles on α-synuclein aggregation, as depicted in Figure 5. The results showed that treatment with arimoclomol nanomicelles led to a significant reduction of ThT fluorescence of α-synuclein fibrils compared to the control, suggesting their ability to interfere with the pathological aggregation process of α-synuclein. However, arimoclomol solubilized in DMSO shows only a weak effect on α-synuclein disaggregation, significantly lower than that observed for the fibril-destabilizing effect of arimoclomol on Aβ_1–42_ fibrils. Furthermore, arimoclomol nanomicelles efficiently inhibited the aggregation of α-synuclein monomer, with almost 100% inhibition at a concentration of 200 μM, while nanomicelles alone exhibited only a small effect. These findings suggest that arimoclomol nanomicelles exhibit antifibrillogenic activity on both Aβ_1–42_ and α-synuclein, the latter being more sensitive to the inhibitory effect of arimoclomol nanomicelles compared to Aβ_1–42_. Given that α-synuclein aggregation is a hallmark of Parkinson’s disease, these findings suggest that arimoclomol nanomicelles have the potential to inhibit this pathological process, further underscoring their therapeutic promise for treating neurodegenerative disorders such as Parkinson’s disease.

### 3.5 In Vivo Biodistribution: Tissue Deposition

To evaluate the distribution profile of arimoclomol nanomicelles, we conducted biodistribution studies using [^99^mTc] radiolabeling. The radiolabeling efficiency was assessed via chromatographic analysis, and the data presented in Table 1 indicate a labeling efficiency exceeding 90% at all evaluated time points. This high labeling efficiency suggests that the [^99^mTc]-labeled arimoclomol nanomicelles were effectively prepared, enabling accurate tracking and assessment of their biodistribution in subsequent studies.

**Table 1:** Quality Control of Arimoclomol Nanomicelles Radiolabeling

parameters quality control						
time [h]	0	1	2	4	6	22
mean [%]	100	99.4	99.7	99.7	99.7	99.2
std. error of mean	0	0.05	0.03	0.03	0.05	0.03

**Figure 6:**
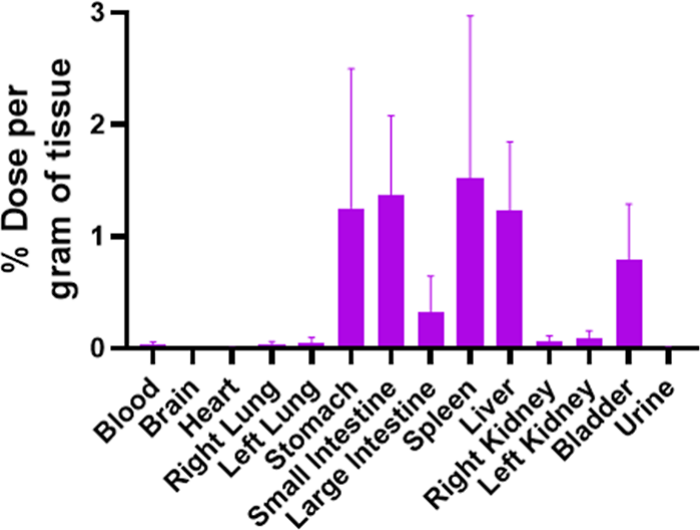
Biodistribution assay in healthy BALB/c mice using 99 mTc radiolabeled arimoclomol nanomicelles. The results were obtained 24 h after intraperitoneal injection from organs such as the brain, heart, lungs, stomach, liver, spleen, kidneys, intestines (large and small) and bladder, in addition to blood and urine. The graph was performed using the standard error of the mean (SEM).

Next, we evaluated the arrangement of the arimoclomol nanomicelles in the organs 24 h after injection. We observed consistent activity in organs (Figure 6) such as the stomach, small and large intestines, spleen, liver and bladder. Activity in the heart, brain, kidneys and lungs was not evident.

**Figure 7:**
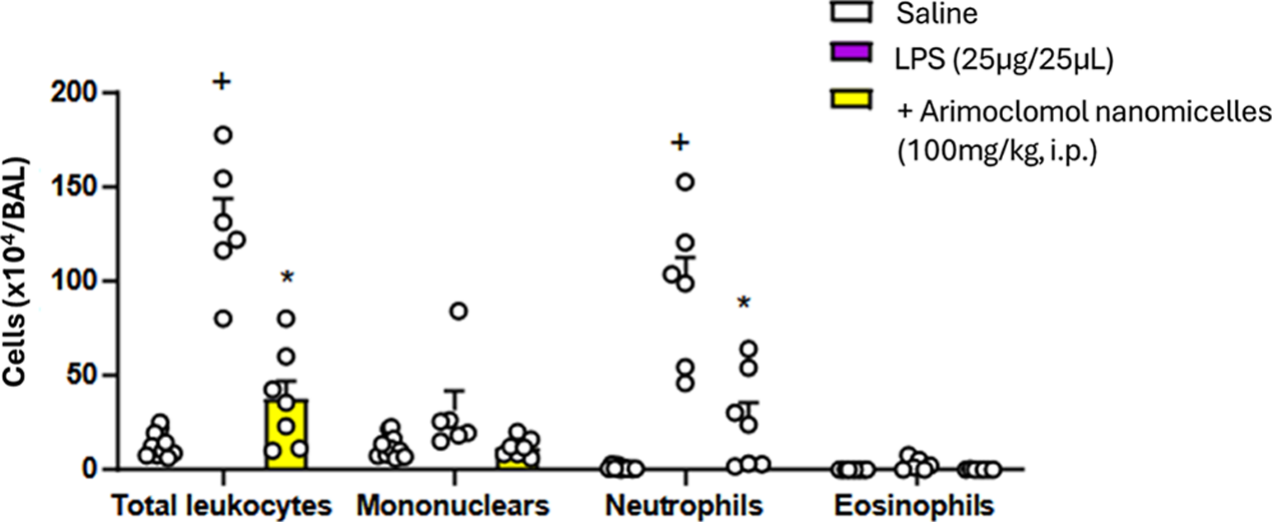
Effect of treatment with arimoclomol nanomicelles (100 mg/kg, i.p.) on LPS-induced leukocyte changes (total and differential cell counts) in the BALF. The bronchoalveolar lavage fluid from C57BL/6 mice was collected 24 h after LPS (25 μg) stimulation. Values are mean ± SEM from at least 6 animals. +*P* < 0.05 as compared with saline-stimulated mice. *P < 0.05 as compared with LPS-stimulated mice.

Challenge of Swiss–Webster mice with LPS (25 μg/25 μL) led to a marked increase in the total leukocyte counts in the BALF, which was accounted for by a massive increase in the number of neutrophils. These changes were clearly sensitive to treatment with arimoclomol nanomicelles (100 mg/kg, i.p.) (Figure 7).

### 3.6 Pharmacokinetic Assay

**Figure 8:**
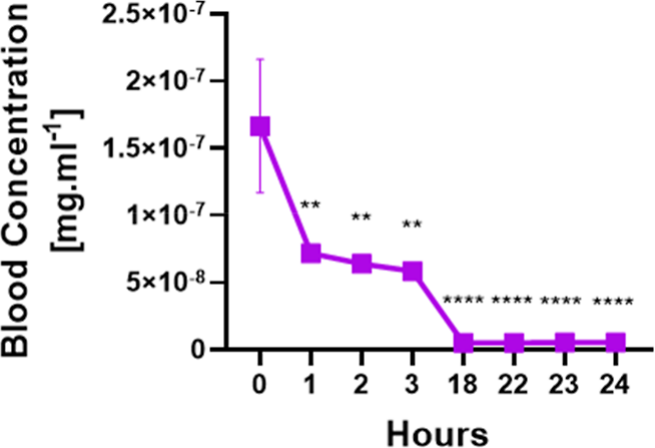
Pharmacokinetic test to evaluate the permanence of arimoclomol nanomicelles in the blood. Graph expressed as blood concentration per time. Ordinary one-way ANOVA was used (SEM). **P < 0.01 and ****p < 0.0001 were considered significant.

To evaluate the pharmacokinetic profile and bloodstream permanence of [99 mTc]-labeled arimoclomol nanomicelles, we conducted pharmacokinetic tests, as shown in Figure 8. Blood samples (2 μL) were collected from the tail vein at various time points following intraperitoneal injection. The results revealed a high concentration of nanomicelles immediately postinjection (time 0 h), followed by a gradual decline over the first 1–3 h. By 18 to 24 h, the presence of nanomicelles in the bloodstream had dropped to extremely low levels, indicating that the nanomicelles have a relatively short circulation time. These findings suggest that while the arimoclomol nanomicelles demonstrate initial rapid distribution, their clearance from the bloodstream is efficient, which could have implications for optimizing dosing regimens in therapeutic applications.

In addition to assessing the pharmacokinetic profile via blood sampling, we evaluated several key parameters, as summarized in Table 2. These parameters include the concentration at time 0, the elimination rate constant, the volume of distribution (*V*_d_), the elimination half-life (*t*_1/2_), and clearance (CL) of the [^99^mTc]-labeled arimoclomol nanomicelles (Table 2). These data indicate that the arimoclomol nanomicelles have a moderate distribution volume, suggesting they primarily remain within the vascular compartment with some tissue distribution. The elimination of half-life of approximately 5.1 h signifies a relatively sustained presence in the body before clearance. The clearance rate, being quite low, implies that the nanomicelles are eliminated at a slow rate, further suggesting the potential for sustained therapeutic effects. This pharmacokinetic profile provides valuable insights into the behavior of arimoclomol nanomicelles in vivo, which is crucial for optimizing their dosing and therapeutic application in neurodegenerative disease treatment.

**Table 2:** Pharmacokinetic Profile of Arimoclomol Nanomicelles

parameters	concentration at zero-time (Co) (mg/mL)	elimination rate/elimination constant (*k*) (mg/h)	volume of distribution (mL)	elimination half-life (1/2) (h)	clearance (L/h)
mean	9.568 × 10^–8^	0.1365	1.067	5.112	0.00015
SEM	7.679 × 10^–9^	0.007	0.08	0.29	3.440 × 10^–6^

### 3.7 Biochemistry Analysis

To complete the in vivo experiments, in Table 3, healthy mice were given arimoclomol nanomicelles intraperitoneally. After 24 h, biochemical parameters were measured. From a hepatic perspective, no changes were observed in the ALT and GGT parameters, only in AST. No changes were observed in CRE when compared to the untreated animal. In addition, we also assessed LDH-P, CHOL, LPS and GLU, components which also showed no changes compared to the control.

**Table 3:** Biochemical Analysis of Healthy Animals 24 h after Treatment

parameters	mean ± SEM	
	control	treated
ALT (U/L)	187.6 ± 120.8	45.2 ± 13.1
AST (U/L)	0.3 ± 0.1	55.8 ± 19.5
GGT (U/L)	15.1 ± 9.6	14.9 ± 10.2
CRE (mg/dL)	0.0 ± 0.0	0.0 ± 0.0
LDH-P (mg/L)	635.7 ± 345.8	924.2 ± 429.5
CHOL (mg/dL)	95.1 ± 11.5	34.4 ± 3.2
LPS (mg/dL)	2400 ± 1417	2177 ± 296.1
AMS (U/L)	200^30^	302 ± 27.1
GLU (mg/dL)	111.2 ± 9.8	54.9 ± 17.2

## 4 Discussion

The release profile results demonstrated an initial increase in arimoclomol release within the first few hours, followed by a sustained release throughout the duration of the experiment. A sustained release system, as observed here, is characteristic of a drug delivery mechanism designed to maintain a prolonged therapeutic effect by gradually releasing the drug over an extended period, which can span from days to months after administration. This gradual and consistent release is advantageous in maintaining stable drug concentrations, reducing dosing frequency, and enhancing therapeutic efficacy, making arimoclomol nanomicelles a promising candidate for long-term treatment of neurodegenerative diseases.^31^

The application of polymeric nanoparticles and nanomicelles are highly effective for drug delivery to the central nervous system (CNS) due to several advantageous properties, including their small size, which facilitates crossing the blood–brain barrier (BBB), their specific targeting and sustained drug release, as well as their inherent biocompatibility, ensuring minimal toxicity.^32,33^ Additionally, these nanocarriers are responsive to various physiological stimuli (e.g., pH, temperature, or enzymatic activity), allowing for controlled and targeted drug release. Furthermore, their surfaces can be customized with ligands or targeting molecules, enabling precise delivery to specific CNS cells or receptors, making them versatile platforms for various biomedical applications, especially in the treatment of neurodegenerative disorders.^34^

The initial characterization of arimoclomol nanomicelles using atomic force microscopy revealed an average size of 552 nm. Studies have demonstrated that the transport efficiency of nanoparticles (NPs) through endothelial cells, such as those forming the blood–brain barrier (BBB), is highly dependent on particle size. Specifically, spherical nanoparticles with a diameter of 200 nm exhibit a permeability that is three times greater than that of 100 nm nanoparticles. In contrast, nanoparticles with a diameter of 500 nm display significantly reduced permeability—100 times lower than 200 nm particles and 10 times lower than 100 nm particles. This suggests that the 552 nm nanomicelles might face challenges in penetrating the BBB efficiently, indicating that further optimization in reducing nanomicelle size could enhance their permeability and effectiveness for CNS drug delivery.^35^ In Raman spectroscopy analysis a peak at 2500 cm^–1^ is unusual as it does not normally correlate with common vibrational modes (e.g., C–H, O–H or N–H stretching). The broadening of the peak for free arimoclomol and its sharpening in nanomicelles suggests a change in the molecular environment or interaction after encapsulation (Supporting Information).

The biodistribution assay revealed significant uptake of arimoclomol nanomicelles in the stomach, intestines, spleen, liver, and bladder. The elevated uptake in the stomach is likely due to the intraperitoneal (I.P.) route of administration, which often leads to prolonged accumulation in the abdominal cavity.^36^ The high uptake by the liver and spleen can be attributed to the mononuclear phagocyte system (MPS),^37^ which actively captures and processes nanoparticles, especially those administered intraperitoneally. Lastly, the observed uptake in the kidneys and bladder indicates renal clearance, suggesting that a portion of the nanomicelles is eliminated through the urinary system. This biodistribution profile is consistent with the expected pathways of nanoparticle uptake and clearance, providing valuable insights into the pharmacokinetic behavior of arimoclomol nanomicelles. These biodistribution data are consistent with the blood concentration findings, where a significant decline in nanomicelles concentration was observed within the first hour postadministration, with this reduction remaining steady throughout the study period. This rapid decrease aligns with the calculated elimination half-life of just a few hours, indicating that the nanomicelles are swiftly cleared from systemic circulation. Additionally, the observed clearance rate of 0.00015 L (150 μL) per hour further supports the efficient elimination process, likely driven by renal clearance and uptake by organs such as the liver and spleen via the mononuclear phagocyte system (MPS). Together, these data provide a comprehensive understanding of the pharmacokinetic behavior and clearance profile of arimoclomol nanomicelles in vivo.

The biochemical analysis was conducted to assess the potential toxicity of the arimoclomol nanomicelles. We evaluated the activity of several key enzymes and biomarkers, including alanine aminotransferase (ALT), aspartate aminotransferase (AST), gamma-glutamyl transferase (GGT), creatinine (CRE), lactate dehydrogenase (LDH-P), cholesterol (CHOL), lipase (LPS), amylase (AMS), and glucose (GLU). The aminotransferases ALT and AST are critical biomarkers for liver damage, as they are involved in gluconeogenesis by catalyzing the transfer of amine groups from alanine and aspartic acid to ketoglutaric acid, producing pyruvic acid and oxaloacetic acid, respectively. Typically, in liver disease, ALT activity is higher than AST since both enzymes are predominantly located in the cytosol of hepatocytes. Elevated levels in the bloodstream indicate hepatocellular injury, though not necessarily cell death.^38^

Our data revealed an increase in ALT activity without a corresponding rise in AST, suggesting mild hepatocellular stress or injury associated with the presence of arimoclomol nanomicelles, as confirmed by the biodistribution assay showing nanomicelle accumulation in the liver. However, the GGT enzyme showed no significant changes compared to the control, suggesting no cholestatic or biliary damage.

No alterations were observed in creatinine levels, indicating no renal impairment. Similarly, there were no changes in LDH-P or pyruvate, suggesting that anaerobic metabolism remains unaffected.^39^ The activities of lipase (LPS) and amylase (AMS) remained stable, indicating no pancreatic dysfunction or alterations in fat metabolism.^40,41^ Interestingly, an increase in cholesterol (CHOL) and glucose (GLU) levels was observed. This aligns with findings reported by Wout and colleagues, who noted that animals administered Pluronic via the intraperitoneal (I.P.) route exhibited sustained hypercholesterolemia and hypertriglyceridemia, likely due to stimulation of 3-hydroxy-3-methylglutaryl-coenzyme A (HMG-CoA) reductase in the liver induced by the polymer.^42^

The results presented reinforce the therapeutic potential of arimoclomol nanomicelles, as they act as a coinducer of heat shock proteins (HSPs), increasing the production of Heat Shock Protein 70 (HSP70), aligning with previous studies.^23,24,43^ The induction of HSP70 is crucial for preventing protein aggregation and protecting neuronal cells, as corroborated by data demonstrating its efficacy in contexts of cellular stress and neurodegenerative pathologies.^43^

The arimoclomol nanomicelles effectively reduce thioflavin T fluorescence, which is a critical indicator of the presence of β-amyloid (Aβ_1–42_) and α-synuclein (α-syn) aggregates. This reduction in thioflavin T detection suggests that the nanomicelles significantly decrease the aggregation of both Aβ_1–42_ and α-syn over time. These findings indicate that arimoclomol nanomicelles have a strong potential to disrupt or inhibit the formation of these pathological protein structures, highlighting their therapeutic promise for targeting key pathological processes in Alzheimer’s and Parkinson’s diseases.^44^ This indicates a significant decrease in both Aβ_1–42_ and α-synuclein aggregates over time, suggesting that the arimoclomol nanomicelles are highly effective in reducing the formation and accumulation of these pathological structures.

The efficiency of arimoclomol nanomicelles in the aggregation process is remarkable. However, there was also a brief reduction in the disaggregation of β amyloid peptides when exposed to the polymer. It was observed that polymeric nanoparticles can modulate the behavior of Aβ peptides, establishing an equilibrium rate, binding to them in the conformation of monomers and oligomers, reducing the fibrillation process.^34,45^ Considering this, Chakraborty and colleagues (2023) have shown that nanoparticles based on copolymers with varying hydrophobicity can promote an antifibrillation effect,^46^ this may indicate the effect observed in our results.

The induction of the activation of heat shock proteins by arimoclomol has recently been associated with a reduction in the cytotoxic effects caused by poorly coiled and aggregated proteins such as β amyloid and Tau protein due to the activation of the heat shock response pathway (HSP).^47^ In cellular mechanisms, the interaction of chaperones and problematic proteins leads to their degradation.^48^ The direct chemical mechanism of the interaction between arimoclomol and the fibers or monomers is still unclear, although the brief reduction observed in Figure 4B is unclear. However, our results show that arimoclomol encapsulated in nanomicelles has a noticeable effect on both mature fibrils and monomers at the highest concentration of 200 μM, an effect that is not fully observed when comparing it to arimoclomol alone or to empty nanomicelles.

Unfortunately, we are still unsure about the mechanisms underlying the interaction between the empty Pluronic F-127 nanomicelle and α-synuclein. However, a possible explanation can be found in the study by Salay and collaborators (2018). They demonstrated that Pluronic in aqueous solution can interact with peptides, such as the antimicrobial peptide tryptricin (TRP3), forming stable F127-TRP3 complexes.^49^ This interaction is attributed to the amphiphilic structure of F127, composed of the PEO–PPO–PEO chain, which favors the formation of associations with specific molecules. These data may help explain the reduction in ThT fluorescence observed in our study.

In these experiments, we used preformed fibrils of α-Syn (produced in the presence of 5 μM ThT) and the decay of ThT fluorescence in the begging of kinetics is explained by the dilution of α-Syn fibrils (4-fold dilution), which is expected to cause their partial dissociation. It is worth noting that the concentration of ThT during this step is kept exactly in 5 μM, which means there is no change in ThT concentration before and after the dilution of fibrils and there is probably no effect of ThT favoring the formation of specific fibrils with a structure different from what we had at the beginning. During the incubation of the mixture, we observed an enhancement of ThT fluorescence after ∼200–300 min due to the reaggregation of the fibrils.

On the other hand, we decided to evaluate the influence of arimoclomol nanomicelles in an animal model of acute inflammation, where the animals were induced with LPS and leukocytes, mononuclear cells, neutrophils and eosinophils were counted in the lung lavage. Arimoclomol nanomicelles acts to reduce both total leukocytes and neutrophils. During the acute inflammatory process, leukocytes are initially recruited, especially neutrophils, ensuring an adequate response to inflammatory agents.^50^ This robust inflammatory response induced by LPS targets the recognition of the myeloid differentiation protein (MD2), Toll receptor (TLR4) and the differentiation cluster macromolecular complex (CD14).^51^

It is possible that arimoclomol nanomicelles influences the negative regulation of pro-inflammatory cytokines, such as IL-1β, IL-6, and TNF-α, by modulating the inflammatory response mediated by the TLR4 pathway and other intracellular inflammatory pathways, significantly reducing the total leukocyte and neutrophil count, indicative of immune response modulation. The regulation occurs, in part, through the inhibition of NF-κB activation, a key transcription factor in the production of pro-inflammatory cytokines. With the suppression of NF-κB signaling, there is a reduction in the expression of IL-1β, IL-6, and TNF-α, cytokines directly involved in amplifying the inflammatory response.^52,53^

Nanomaterials such as lipid nanoparticles (liposomes and solid lipid NPs), nanoemulsions and polymeric nanoparticles (polymeric NPs, dendrimers, nanogels and micelles)^54^ are considered innovative components to enrich the field of anti-inflammatory therapies, due to their unique physicochemical characteristics.^55^ The impact of inflammation as an underlying mechanism in the progression of diseases such as Alzheimer’s and Parkinson’s reinforces the relevance of the anti-inflammatory properties observed with arimoclomol nanomicelles.^56^ Work has shown that nanosystems can reduce inflammation by inducing the polarization of macrophages toward the M2 phenotype, reducing the expression of pro-inflammatory cytokines, thus blocking leukocyte adhesion and ultimately reducing oxidative stress.^57−59^ Therefore, these results indicate a one-off reduction in the inflammatory process mediated by arimoclomol nanomicelles which suggests a modulation like nanoceria and Se@SiO_2_, that can modulate of the NF-κB and Nrf2 signaling pathway, leading to a reduction in inflammation.^60^ Or even integration into combined therapeutic strategies, such as the use of antioxidant agents enhancing neuroprotection by reducing oxidative stress and modulating protein aggregation, thereby increasing the efficacy in treating diseases like Alzheimer’s and Parkinson’s, according to some studies.^60,61^

All results highlight the efficiency of the nanomicelles as a potential therapeutic strategy for targeting protein aggregation in neurodegenerative diseases such as Alzheimer’s and Parkinson’s, but it also sheds light on the treatment of inflammatory diseases.

## 5 Conclusions

Arimoclomol nanomicelles offer a promising solution for long-term treatment of neurodegenerative diseases like Alzheimer’s and Parkinson’s due to their sustained release, which maintains stable drug concentrations and reduces the need for frequent dosing. Biodistribution studies showed significant uptake in the liver, spleen, and kidneys, with renal clearance being the primary elimination route. Although mild hepatocellular stress was observed, no significant liver or kidney damage occurred. Additionally, the nanomicelles demonstrate anti-inflammatory properties by reducing leukocyte and neutrophil counts, highlighting their potential for treating both neurodegenerative and inflammatory diseases.

## Data Availability

All data will be available under request.

## Supporting Information Available

Detailed discussion on the interpretation of the Raman spectra of arimoclomol and its nanomicelles, highlighting changes in molecular interactions after encapsulation, such as the reduction of hydrogen bonding and its impact on peak broadening and sharpness. Additionally, it explores the influence of fluorescence on the results and the importance of baseline correction for a more accurate analysis of the observed Raman peaks (PDF)
