# Supplementary material for: Therapeutic Potential of Arimoclomol Nanomicelles: In Vitro Impact on Alzheimer’s and Parkinson’s Pathology and Correlation with In Vivo Inflammatory Response
Source: ACS Chem Neurosci. 2025 Feb 5;16(4):699–710. doi: 10.1021/acschemneuro.4c00734 (PMC11843614; doi:10.1021/acschemneuro.4c00734)
Supplement: Supplementary file 1 — cn4c00734_si_001.pdf [file cn4c00734_si_001.pdf]

SUPPLEMENTARY MATERIALS

Manuscript ID: cn-2024-007349

Therapeutic Potential of Arimoclomol Nanomicelles: *In Vitro* Impact on Alzheimer's and Parkinson's Pathology and Correlation with *In Vivo* Inflammatory Response

Isabelle Xavier-de-Britto[1], Natália Cristina Gomes-da-Silva[1], Marilia Amável Gomes Soares[1], Cristian Follmer[2], David Dabkiewicz[2], Luciana Magalhães Rebelo Alencar[3], Celso Sant'Anna[4], Tatiana Paula Teixeira Ferreira[5], Patrícia Machado Rodrigues e Silva Martins[5], Eduardo Ricci-Junior[6], Pierre Basílio Almeida Fechine[7], Ralph Santos-Oliveira[1,8]

1- Brazilian Nuclear Energy Commission, Nuclear Engineering Institute, Laboratory of Nanoradiopharmacy and Synthesis of New Radiopharmaceuticals, Rio de Janeiro 21941906, RJ, Brazil

2- Laboratory of Biological Chemistry of Neurodegenerative Disorders, Department of Physical Chemistry, Institute of Chemistry, Federal University of Rio de Janeiro, Rio de Janeiro 21941-909, Brazil.

3- Biophysics and Nanosystems Laboratory, Federal University of Maranhão, Department of Physics, São Luis 65065690, MA, Brazil

4- Laboratory of Microscopy Applied to Life Science - Lamav, National Institute of Metrology, Quality and Technology, Duque de Caxias, RJ, Brazil

5- Laboratory of Inflammation, Oswaldo Cruz Institute, Oswaldo Cruz Foundation, Rio de Janeiro 21040-360, Brazil

6- Federal University of Rio de Janeiro, School of Pharmacy, Rio de Janeiro, 21941900, RJ, Brazil
7- Group of Chemistry of Advanced Materials (GQMat)- Department of Analytical Chemistry and Physical-Chemistry, Federal University of Ceará, Fortaleza-CE, 451-970, Brazil

8- Rio de Janeiro State University, Laboratory of Radiopharmacy and Nanoradiopharmaceuticals, Rio de Janeiro 23070200, RJ, Brazil.

All correspondence to:

Dr. Ralph Santos-Oliveira

Brazilian Nuclear Energy Commission

Nuclear Engineering Institute

Rio de Janeiro/RJ, Brazil

Rua Hélio de Almeida, 75, Ilha do Fundão-Rio de Janeiro-Brazil

E-mail: roliveira@ien.gov.br

S1. RESULTS

Raman Spectroscopy

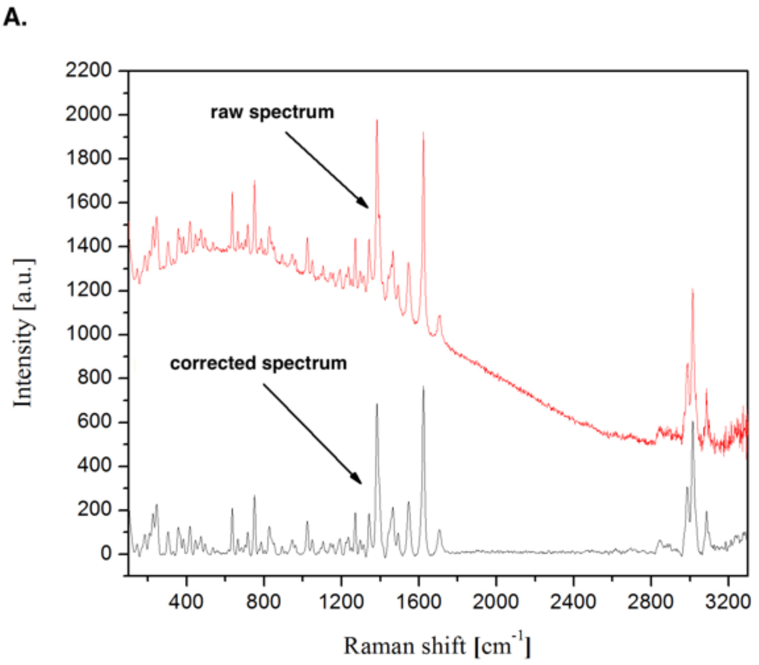

Figure S1: Baseline correction of Raman spectra. Baseline subtraction effectively removes the broad fluorescence background, leaving only true Raman peaks for analysis, eliminating the influence of fluorescence.

S2. DISCUSSION

Raman Spectroscopy

In Raman spectroscopy, a peak at 2500 cm^{-1} is unusual because it does not normally correlate with common vibrational modes (e.g., C-H, O-H, or N-H stretching). The broadening of the peak for free arimoclomol and its sharpening in nanomicelles suggests a change in the molecular environment or interaction after encapsulation.

In Raman spectroscopy, the observed frequency (ν\nuv) of a vibrational mode can be expressed as:

$$\nu = \frac{1}{2\pi c}\sqrt{\frac{k}{\mu}}$$

where:

c is the speed of light,

k is the bond force constant,
μ is the reduced mass of the vibrating atoms.

A broad peak at 2500 cm^{-1} may indicate weak hydrogen bonding, which affects the vibrational energy of functional groups such as O-H. When encapsulated, a change in the local environment can reduce hydrogen bonding, increasing the strength constant k, which sharpens the peak. The sharpness of the peak at 2500 cm^{-1} after encapsulation likely indicates a change in intermolecular forces, such as reduced hydrogen bonding. The absence of the OH peak requires verification that the spectrum captures the correct range.

An alternative explanation could be the influence of fluorescence on the analysis, as when using a 532 nm laser, fluorescence can be a common problem, particularly for organic compounds such as arimoclomol or its impurities. Fluorescence appears as a broad, featureless background in Raman spectra, which can obscure the actual Raman active peaks.

After encapsulation into nanomicelles, the molecular environment changes, potentially leading to quenching of the fluorescence. It is important to note that quenching reduces the intensity of the fluorescence signal, which results in the spectrum appearing sharper and the Raman peaks becoming more distinguishable. Typical vibrational modes for functional groups occur in well-defined regions: O-H stretching: 3200–3600 cm^{-1}. C-H stretching: ~2900–3100 cm^{-1}, C≡C and C≡N stretching: ~2100–2300 cm^{-1}

There is no common functional group associated with a vibrational mode at 2500 cm^{-1}. Therefore, attributing this broad feature to a Raman-active mode is unlikely. Therefore, appropriate baseline subtraction was performed to effectively remove the broad fluorescence background, leaving only true Raman peaks for analysis. Thus, by correcting the baseline, as illustrated in Figure S1, only the Raman peaks are highlighted, eliminating the influence of fluorescence and facilitating data interpretation.
